# Mode of Detecting Small Quantities of Opium

**Published:** 1848-11

**Authors:** 


*Mode of Detecting Small Quantities of Opium.*—The process proposed by M. Hensler consists in treating the powder of opium by boiling sulphuric ether, and evaporating; a fat, viscous residue is left, containing crystals of meconine and narcotine. If this compound be treated with boiling water, the meconine is dissolved, and the narcotine may then be dissolved in alcohol; but in the solution thus procured another substance exists, named by Merk porphyroxine, which possesses the property of assuming a reddish purple colour if heated with dilute hydrochloric acid. Porphyroxine is neutral, and crystallizes in brilliant needles. Sulphuric and nitric acids render it of an olive colour; it is dissolved in dilute sulphuric muriatic acids, and the heat produces a rosy or reddish purple colour, according to the degree of dilution. Alkalies decolorize the liquid, and throw down a white precipitate.

If it is wished to discover opium in a compound medicinal preparation, a little liquor potassæ should be first added, and then some ether should be stirred in; after that, a strip of blotting-paper should be soaked in the ethereal extract, and then dried, repeating this several times. If the strip be now moistened with dilute hydrochloric acid, and brought into contact with watery vapour, it becomes more or less red, in proportion to the quantity of opium present.

Porphyroxine (which gives this characteristic reaction) being insoluble in water, the above will furnish no test result with preparations of opium, having only the gummy (or watery extract) for their base; crude opium itself, or an alcoholic or ethereal solution of it, must be present, in order to the efficacy of the test.—*Ibid.*

